# New uracil analog U-332 is an inhibitor of NF-κB in 5-fluorouracil-resistant human leukemia HL-60 cell line

**DOI:** 10.1186/s40360-020-0397-4

**Published:** 2020-03-02

**Authors:** Angelika Długosz-Pokorska, Marlena Pięta, Jacek Kędzia, Tomasz Janecki, Anna Janecka

**Affiliations:** 1 Department of Biomolecular Chemistry, Medical University of Lodz, Mazowiecka 6/8, 92-215 Lodz, Poland; 2 Institute of Organic Chemistry, Faculty of Chemistry, Lodz University of Technology, Lodz, Poland

**Keywords:** Multidrug resistance, NF-κB subunits, Real-time PCR, ELISA assay, Uracil analog U-332

## Abstract

**Background:**

5-Fluorouracil (5-FU) is an antimetabolite that interferes with DNA synthesis and has been widely used as a chemotherapeutic drug in various types of cancers. However, the development of drug resistance greatly limits its application. Overexpression of ATP-binding cassette (ABC) transporters in many types of cancer is responsible for the reduction of the cellular uptake of various anticancer drugs causing multidrug resistance (MDR), the major obstacle in cancer chemotherapy. Recently, we have obtained a novel synthetic 5-FU analog, U-332 [(*R*)-3-(4-bromophenyl)-1-ethyl-5-methylidene-6-phenyldihydrouracil], combining a uracil skeleton with an *exo*-cyclic methylidene group. U-332 was highly cytotoxic for HL-60 cells and showed similar cytotoxicity in the 5-FU resistant subclone (HL-60/5FU), in which this analog almost completely abolished expression of the ATP-binding cassette (ABC) transporter, multidrug resistance associate protein 1 (ABCC1). The expression of ABC transporters is usually correlated with NF-κB activation. The aim of this study was to determine the level of NF-κB subunits in the resistant HL-60/5-FU cells and to evaluate the potential of U-332 to inhibit activation of NF-κB family members in this cell line.

**Methods:**

Anti-proliferative activity of compound U-332 was assessed by the MTT assay. In order to disclose the mechanism of U-332 cytotoxicity, quantitative real-time PCR analysis of the NF-κB family genes, *c-Rel, RelA, RelB, NF-κB1,* and *NF-κB2,* was investigated. The ability of U-332 to reduce the activity of NF-κB members was studied by ELISA test.

**Results:**

In this report it was demonstrated, using RT-PCR and ELISA assay, that members of the NF-κB family *c-Rel, RelA, RelB, NF-κB1,* and *NF-κB2* were all overexpressed in the 5-FU-resistant HL-60/5FU cells and that U-332 potently reduced the activity of c-Rel, RelA and NF-κB1 subunits in this cell line.

**Conclusions:**

This finding indicates that c-Rel, RelA and NF-κB1 subunits are responsible for the resistance of HL-60/5FU cells to 5-FU and that U-332 is able to reverse this resistance. U-332 can be viewed as an important lead compound in the search for novel drug candidates that would not cause multidrug resistance in cancer cells.

## Background

The incidence of acute myeloid leukemia (AML) and heterogeneous clonal disorders of hematopoietic progenitor cells are increasing worldwide [1]. There are several therapies which are offered for patients with AML but survival after relapse remains poor, necessitating the search for novel chemotherapeutic candidates. At present, the toxic effect of chemotherapy and the occurrence of secondary malignancies associated with AML are the major drawbacks in the pharmacotherapy of AML. Moreover, the frequent acquisition of multidrug resistance (MDR) phenotype is the additional serious problem in the treatment of AML patients. Neoplastic cells are able to develop many different mechanisms of MDR, such as DNA mutations, cell metabolic changes and very often, overexpression of ATP-binding cassette (ABC) transporters and activation of the nuclear factor κB (NF-κB) [2–4].

**Fig. 1:**
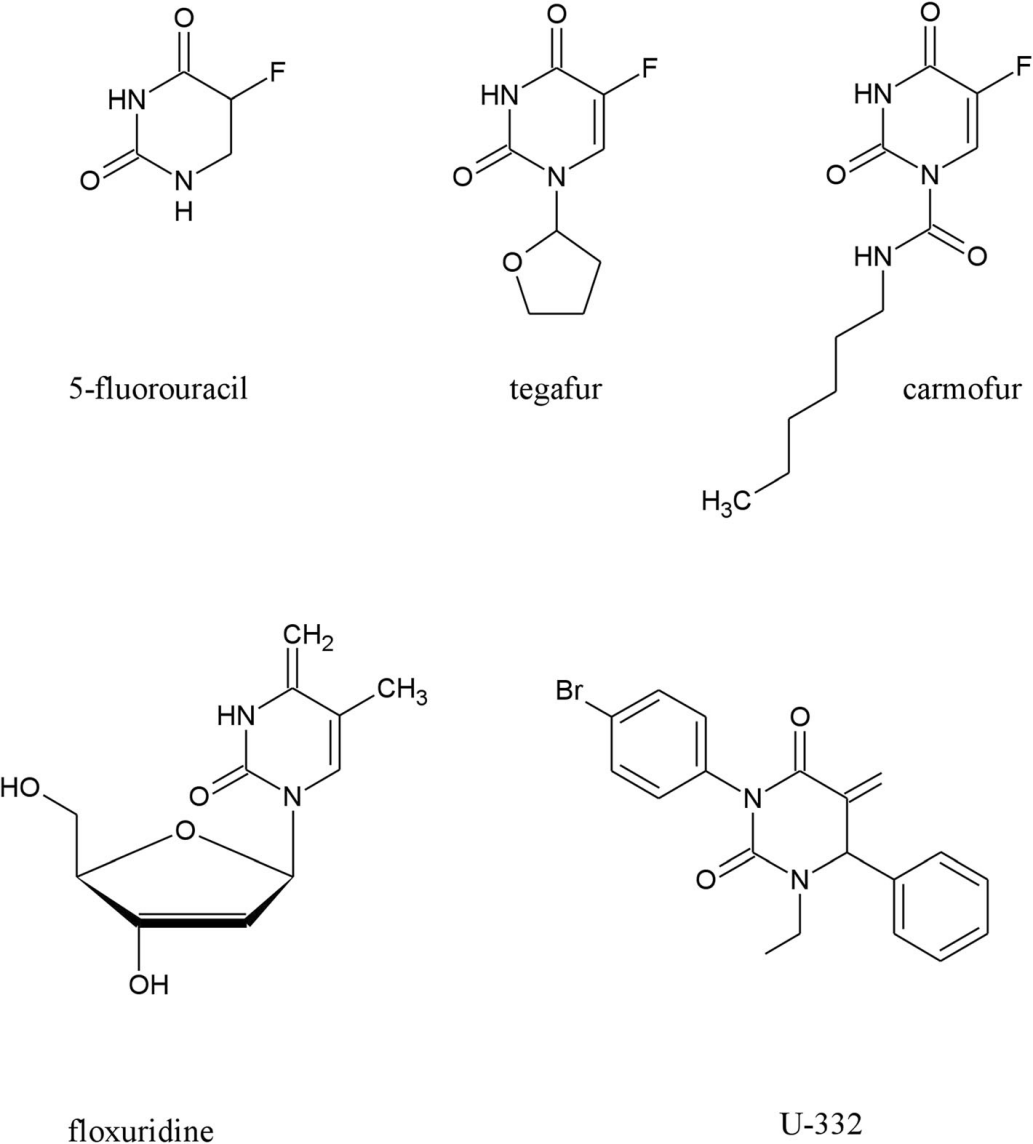
The chemical structure of uracil analogs

5-Fluorouracil (5-FU) was the first synthetic fluoropyrimidine analog that showed pharmacological activity. At the molecular level, 5-FU is an antineoplastic antimetabolite that interferes with DNA synthesis by blocking the thymidylate synthase-catalyzed conversion of deoxyuridylic acid to thymidylic acid [5]. In numerous cancer cells, 5-FU inducesh67hu apoptosis and cell cycle arrest and inhibits proliferation [6–8]. Since its first synthesis in 1957 [9] 5-FU has been widely used as a chemotherapeutic drug in various types of cancer [10]. However, its application is now limited due to the serious side-effects and the development of resistance [11]. Numerous modifications of 5-FU have been proposed so far, and new analogs modified at position 1, such as tegafur, carmofur and floxuridine (Fig. 1) are now replacing 5-FU in clinical practice [12].

Though new anticancer agents continue to be discovered, the problem of resistance is still a major obstacle in obtaining efficient drug candidates. Among various mechanisms that can be developed by neoplastic cells in order to escape medical intervention, overexpression of ATP-binding cassette (ABC) transporters is the one well documented [3, 4]. The ABC transporter family are transmembrane proteins involved in the normal physiological process but also in the mechanism of drug resistance in cancer cells. The ABC proteins function as efflux pumps that can transport drugs out of the cells in the ATP energy-dependent mechanism, reducing their intracellular concentration [13]. The best known transporters that were shown to reduce the cellular uptake of anticancer drugs, including 5-FU, are P-glycoprotein (ABCB1), multidrug resistance associate protein 1 (ABCC1) and breast cancer resistance protein (ABCG2) [14–20].

The mechanism of ABC transporter regulation is still not fully understood. Recently, many transcription factor-binding sequences, such as those for p53, AP-1 and, very often, for NF-κB have been identified in the promoter region of the ABCB1 gene [21–23].

**Fig. 2:**
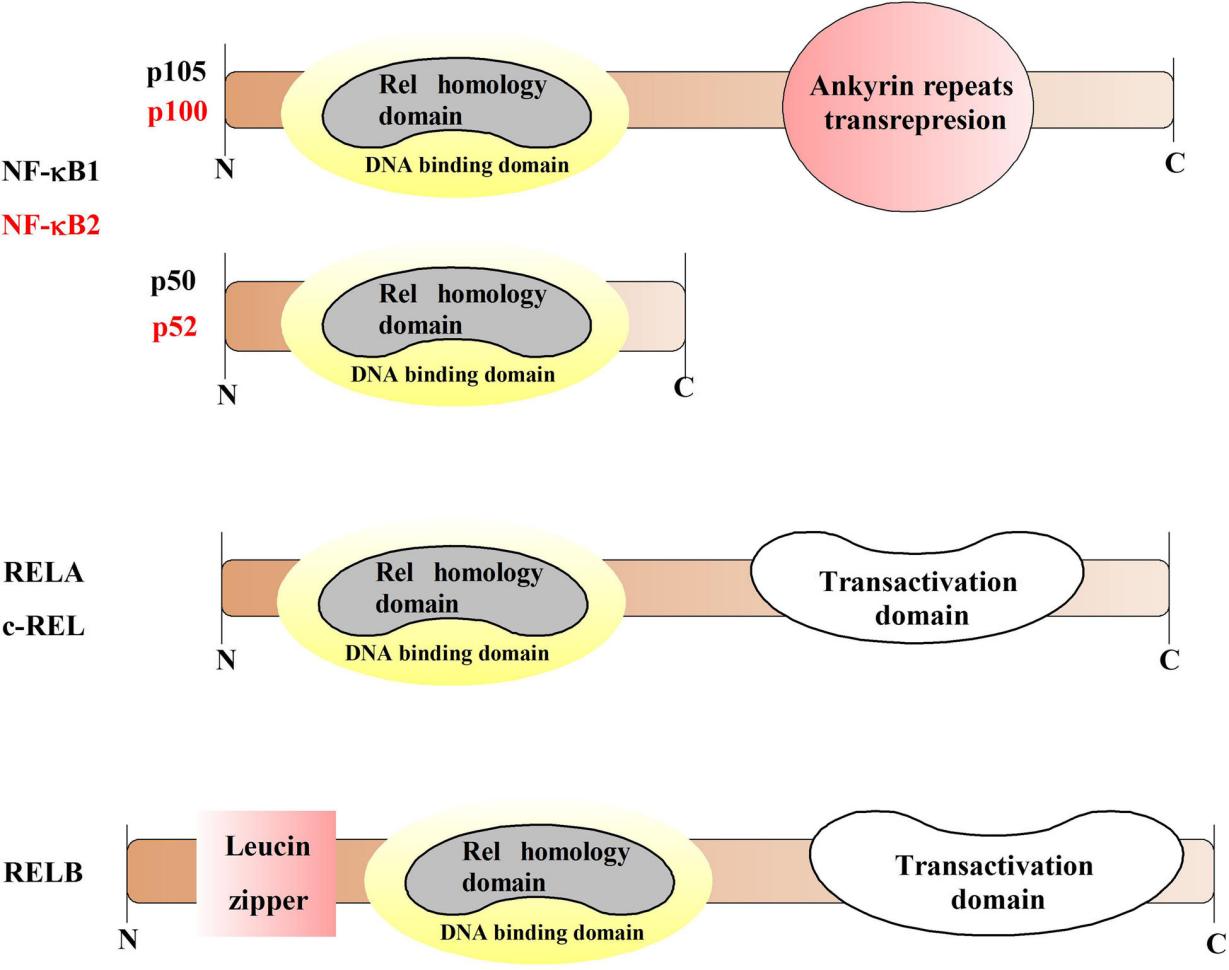
The members of NF-κB family. The Rel homology domain is characteristic for all memb ers the NF-κB family, while the ankyrin repeats only for the NF-κB inhibitors and the NF-κB precursors of p50 and p52 subunits (p100, p105). The transactivation domain is a C-terminal non-homologous domain that is important for *NF*-*κB*-mediated gene transactivation. For activation of transactivation domain, RelB requires an amino-terminal leucine zipper region

NF-κB consists of a family of five proteins, p65 (RelA), RelB, c-Rel, p105/p50 (NF-κB1), and p100/52 (NF-κB2) that may form different transcriptionally active homo- and heterodimeric complexes [24] (Fig. 2). The most important subunit of the NF-κB family is RelA/p65, which is phosphorylated in the posttranslational activation mechanism [24, 25]. RelB is the only NF-κB subunit that does not form homodimers and can trigger a potent transcriptional activation only when coupled to p50 or p52. The c-Rel plays an essential role in the regulation of T-cell-mediated immunity [26]. NF-κB1 and NF-κB2 are the precursor forms of p50 and p52, respectively [27].

Various chemotherapeutic drugs, including 5-FU, were shown to activate NF-κB. The members of the NF-κB family can be activated in several ways. In the classical NF-κB pathway, various cellular receptors, such as tumor necrosis factor receptors (TNFRs), are activated. Then, the inhibitory IκB proteins, that form dimmers with NF-κB subunits, are phosphorylated at two specific serine residues. The IκB activation leads to disintegration of the NF-κB complexes. RelA- and c-Rel-containing dimers translocate to the nucleus where they regulate over 100 transcription targets. The genes whose expression is regulated by NF-κB play an important role in immune/stress responses, apoptosis, proliferation, differentiation and development [28, 29].

Continuing the search for novel compounds with anticancer properties, we have recently described a series of uracil analogs, combining uracil skeleton with an *exo*-cyclic methylidene group conjugated with a carbonyl function [30]. These analogs were all highly cytotoxic against leukemia HL-60 cell line. The most potent analog of the series, (*R*)-3-(4-bromophenyl)-1-ethyl-5-methylidene-6-phenyldihydrouracil, designated U-332 (Fig. 1), caused in HL-60 cells excessive DNA damage which led to the cell cycle arrest in G2/M phase and apoptosis. To determine the activity of U-332 in the resistant cells, we recently selected a 5-FU resistant subclone (HL-60/5FU) of HL-60 cell line by the conventional method of the continuous exposure of the cells to 5-FU up to 0.08 mmol/L concentration [31]. HL-60/5FU cells exhibited a 6-fold enhanced resistance to 5-FU as compared with HL-60 cells. RT-PCR and ELISA assay showed significant overexpression of MDR-related ABC transporters, ABCB1, ABCG2 but especially ABCC1 in the HL-60/5FU, as compared with the parental cell line. U-332 almost completely abolished ABCC1 expression in the resistant HL-60/5FU cells disturbing therefore drug efflux [31].

The aim of this study was to determine the level of NF-κB subunits in the resistant HL-60/5-FU cell line and to evaluate the potential of a novel uracil analog U-332 to inhibit activation of NF-κB family members in this cell line. For comparison, bengamide (BGD) [32], a potent inhibitor of NF-κB activation was included in the research.

## Methods

### Chemistry

Synthesis of (*R*)-3-(4-bromophenyl)-1-ethyl-5-methylidene-6-phenyldihydrouracil (U-332) was performed using Horner-Wadsworth-Emmons methodology, as described elsewhere [33]. Starting 3-(4-bromophenyl)-5-diethoxyphosphoryl-1-ethyluracil **1** was transformed into 3-(4-bromophenyl)-5-dichlorophosphoryl-1-ethyluracil **2** which was next reacted with (*R*)-1-phenylethylamine to yield (*R*,*R*)-3-(4-bromophenyl)-5-di (1-phenylethylamino)phosphoryl-1-ethyluracil (*R*,*R*)-**3**. This compound was used as a Michael acceptor in the reaction with phenylmagnesium chloride and obtained trans-adduct (*R*,*R*)-**4** was separated and purified. Absolute configuration of this compound was confirmed by single crystal X-ray technique. When (*R*,*R*)-**4** was applied in Horner-Wadsworth-Emmons olefination of formaldehyde, (*R*)-3-(4-bromophenyl)-1-ethyl-5-methylidene-6-phenyldihydrouracil U-332 was obtained as pure enantiomer (ee > 99%).

### Materials

BGD was obtained from Tocris Bioscience (Bristol, UK). U-332 and BGD were dissolved in DMSO and diluted in culture medium to obtain less than 0.1% DMSO v/v concentration.

### Cell culture

The human leukemia cell line, HL-60 was purchased from the European Collection of Authenticated Cell Cultures (ECACC) and was cultured in RPMI 1640 plus GlutaMax I medium (Invitrogen, Grand Island, NY, USA) containing 10% fetal bovine serum (FBS) and antibiotics (100 μg/mL streptomycin and 100 U/mL penicillin).

5-FU-resistant HL-60 cell line was obtained by a long-time exposure of HL-60 cells to increasing 5-FU concentrations (0.01–10 mg/L). The process was repeated until the cells were able to tolerate up to 10 mg/L of 5-FU. The detailed description of this procedure was given elsewhere [31].

### Metabolic activity - MTT assay

The determination of anti-proliferative activity of U-332 and BGD was performed by the MTT assay, according to the Mosmann method, as described elsewhere [34].

### Quantitative real-time PCR assay

The expression of NF-κB genes was analyzed by real-time PCR (RT-PCR). Briefly, the HL-60 and HL-60/5FU cells were seeded on the 6-well plates at the optimal cell density (3.0 × 10^5^ cells/well in 3 mL of culture media), treated with the tested compounds at IC_50_ concentration each and left to grow for 24 h. Total RNA was directly extracted from cultured cells, using the Total RNA Mini Kit (A&A Biotechnology, Gdynia, Poland) while Transcriba Kit (A&A Biotechnology, Gdynia, Poland) was used for cDNA synthesis, according to the manufacturer’s procedure.

Amplification of cDNA was performed using Real-Time 2x-PCR SYBR Master Mix (A&A Biotechnology, Gdynia, Poland) and gene specific primers (*RelA, RelB, NF-κB1, NF-κB2* and *c-Rel*) (Table 1) in Stratagene MX3005P QPCR System (Agilent Technologies, Inc. Santa Clara, CA, USA) according to the manufacturer’s protocol. The housekeeping gene, *GAPDH,* was used as an internal reference gene for normalization of qPCR results. The gene expression levels were determined by the 2^-∆∆CT^ method [35].

**Table 1:** Primer sequences for RT-PCR reaction

Gene	Primer sequences	
	Forward primer	Reverse primer
*GAPDH*	GTCGCTGTTGAAGTCAGAGGAG	CGTGTCAGTGGTGGACCTGAC
*RelA*	CGGGATGGCTTCTATGAGG	CTCCAGGTCCCGCTTCTT
*RelB*	GGCCTGGGAGAAGTCAGC	GCTCTACTTGCTCTGCGACA
*c-Rel*	TGAACATGGTAATTTGACGACTG	ACACGACAAATCCTTAATTCTGC
*NF-κB1*	ACCCTGACCTTGCCTATTTG	AGCTCTTTTTCCCGATCTCC
*NF-κB2*	GAACTCCTCCATTGTGGAACC	GAACTCCTCCATTGTGGAACC

### Determination of NF-κB subunit activity by ELISA-based method

The activity of NF-κB family members (p50, p52, p65, c-Rel, RelB) was analyzed in the cellular protein extracts (10 μg) by the ELISA-based method using NF-κB (p50, p52, p65, c-Rel, RelB) Transcription Factor Assay Kit (ABCAM). Briefly, HL-60 and HL-60/5FUres cells were seeded in triplicate into 6-well plates at the optimal cell density (4.5 × 10^5^ cells/well in 3 mL of culture media). Then, U-332 and BGD at IC_50_ concentration each were added and cells were left to grow for 24 h. After incubation, cells were washed 3x in PBS and immediately collected by centrifugation (200×g, 5 min).

The Nuclear Extraction Kit was used in the preparation of nuclear extracts which were then analyzed using NF-κB (p50, p52, p65, c-Rel, RelB) Transcription Factor Assay Kit containing a 96-well plate with immobilized oligonucleotides for NF-κB subunit binding site (5-GGGACTTTCC-3 ´). Active NF-κB heterodimers present in the whole-cell extracts appropriately bind to this oligonucleotide.

For detection of p50, p52, p65, c-Rel or RelB, the primary antibodies recognizing an epitope on these NF-κB subunits were used. The secondary antibodies conjugated to horseradish peroxidase (HRP) provided sensitive colorimetric readout at OD 450 nm. The data were visualized using Flexstation 3.

### Statistical analysis

Statistical analyses and all graphs were prepared using Prism 6.0 (GraphPad Software Inc., San Diego, CA, USA). All data are presented as mean ± SEM. Statistical significance was assessed using one-way ANOVA followed by a post-hoc multiple comparison Student-Newman-Keuls test (for comparisons of three or more groups) or Student’s *t*-test (for comparisons of two groups).*p < 0.05, **p < 0.01, and ***p < 0.001, ****p < 0.0001 were considered significant.

## Results

### MTT-cell viability test

**Fig. 3:**
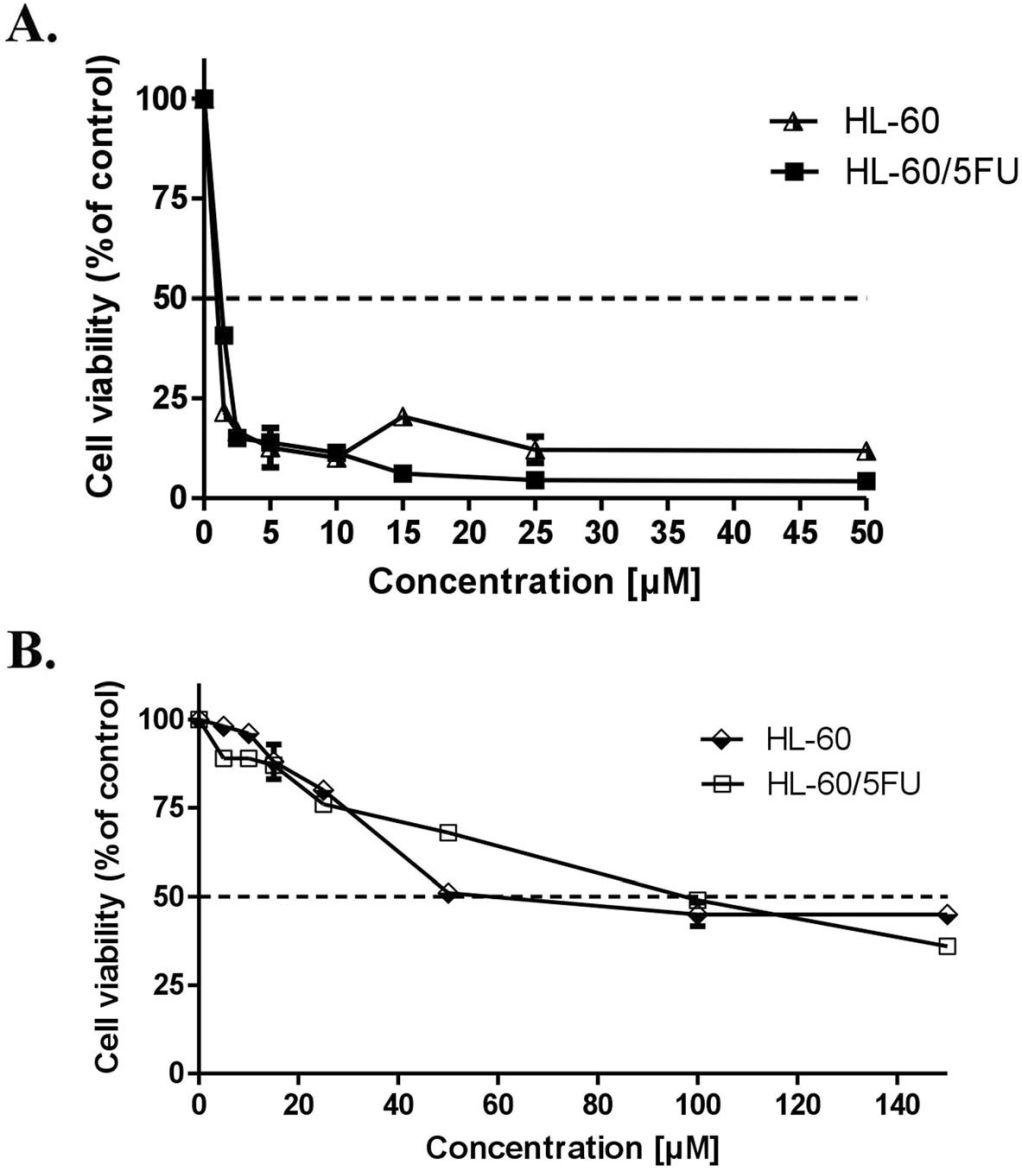
The cytotoxic effect of U-332 (**a**) and BGD (**b**) on HL-60 and HL-60/5FU cells analyzed by MTT assay

Cytotoxic activity of U-332 and BGD was examined using the MTT test. HL-60 and HL-60/5FU cells were exposed to a broad range of compound concentrations for 24 h (Fig. 3). BGD showed only a week cytotoxic effect (IC_50_ = 51 μM and 98 μM in HL-60 and HL60/5FU, respectively), while U-332 was 66- and 110-fold more cytotoxic (IC_50_ = 1.2 μM and 0.9 μM in HL-60 and HL60/5FU, respectively).

### Expression level of NF-κB subunit genes

Analysis of the expression level of *c-Rel*, *RelA, RelB, NF-κB1 (p100/p50)* and *NF-κB2 (p100/p52)* genes in the HL-60 and resistant HL-60/5FU cells was performed by real-time PCR. In the HL-60/5FU cells, relative *RelA, RelB, NF-κB1, NF-κB2* and *c-Rel* mRNA expression levels were 1.1–4.4-fold higher than in the parental HL-60 cells (Table 2).

**Table 2:** Expression of NF-κB subunit genes involved in multidrug resistance in HL-60/5FU cells

gene	*c-Rel*	*RelA*	*RelB*	*NF-κB1*	*NF-κB2*
Control (HL-60)	1.0 ± 0.02	1.0 ± 0.01	1.0 ± 0.08	1.0 ± 0.02	1.0 ± 0.02
HL-60/5FU	4.4 ± 0.2***	2.1 ± 0.1***	1.1 ± 0.04	2.9 ± 0.1***	1.2 ± 0.05

Data represent mean ± SEM of three independent experiments performed in triplicate. ***, *p* < 0.001

**Fig. 4:**
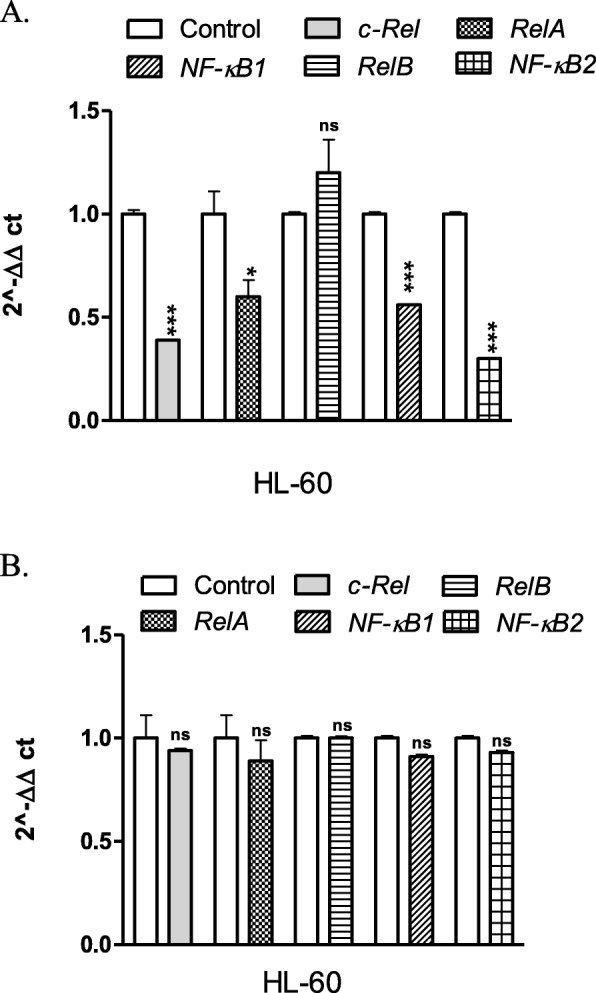
Real-time PCR analysis of *c-Rel, RelA, RelB, NF-κB1 (p100/p50)*, and *NF-κB2 (p100/p52)* mRNA levels in HL-60 cells treated with U-332 (**a**) and BGD (**b**). Data are expressed as mean ± SEM Statistical significance was assessed by *test t*. *p < 0.05, ** *p* < 0.01, *** *p* < 0.001

Then, both types of cells were treated for 24 h with U-332 or BGD (at IC_50_ concentration each). In HL-60 cells, U-332 down-regulated the expression level of *c-Rel*, *RelA, NF-κB1* and *NF-κB2* genes, but the most pronounced effect (3.7-fold) was observed for *NF-κB2* (Fig. 4a). A potent NF-κB inhibitor*,* BGD did not influence *NF-κB* gene expression in this cell line (Fig. 4b).

**Fig. 5:**
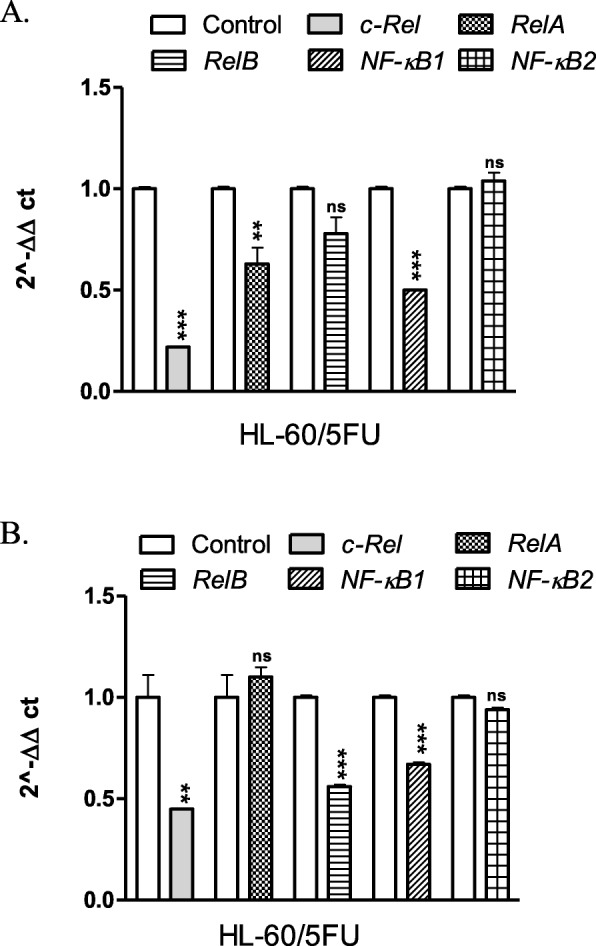
Real-time PCR analysis of *c-Rel, RelA, RelB, NF-κB1 (p100/p50)*, and *NF-κB2 (p100/p52)* mRNA levels in HL-60/5FU cells treated with U-332 (**a**) and BGD (**b**). Data are expressed as mean ± SEM. Statistical significance was assessed by the *test t*. ** *p* < 0.01, *** *p* < 0.001

In the resistant HL-60/5FU cells, U-332 significantly decreased the mRNA level of *c-Rel*, *RelA* and *NF-κB1* (1.7-, 2- and 4.6-fold, respectively), as compared with control (Fig. 5a). BGD down-regulated the expression of *c-Rel*, *RelB* and *NF-κB1* while did not influence *RelA* and *NF-κB2* in these cells (Fig. 5b).

### Activity of NF-κB subunits

To determine the activity of NF-κB family members in HL-60 and HL60/5FU cell lines, the ELISA-based method was used. Cancer cell lysates were prepared after 24 h exposure of cells to analog U-332 or BGD (at IC_50_ concentration each).

**Fig. 6:**
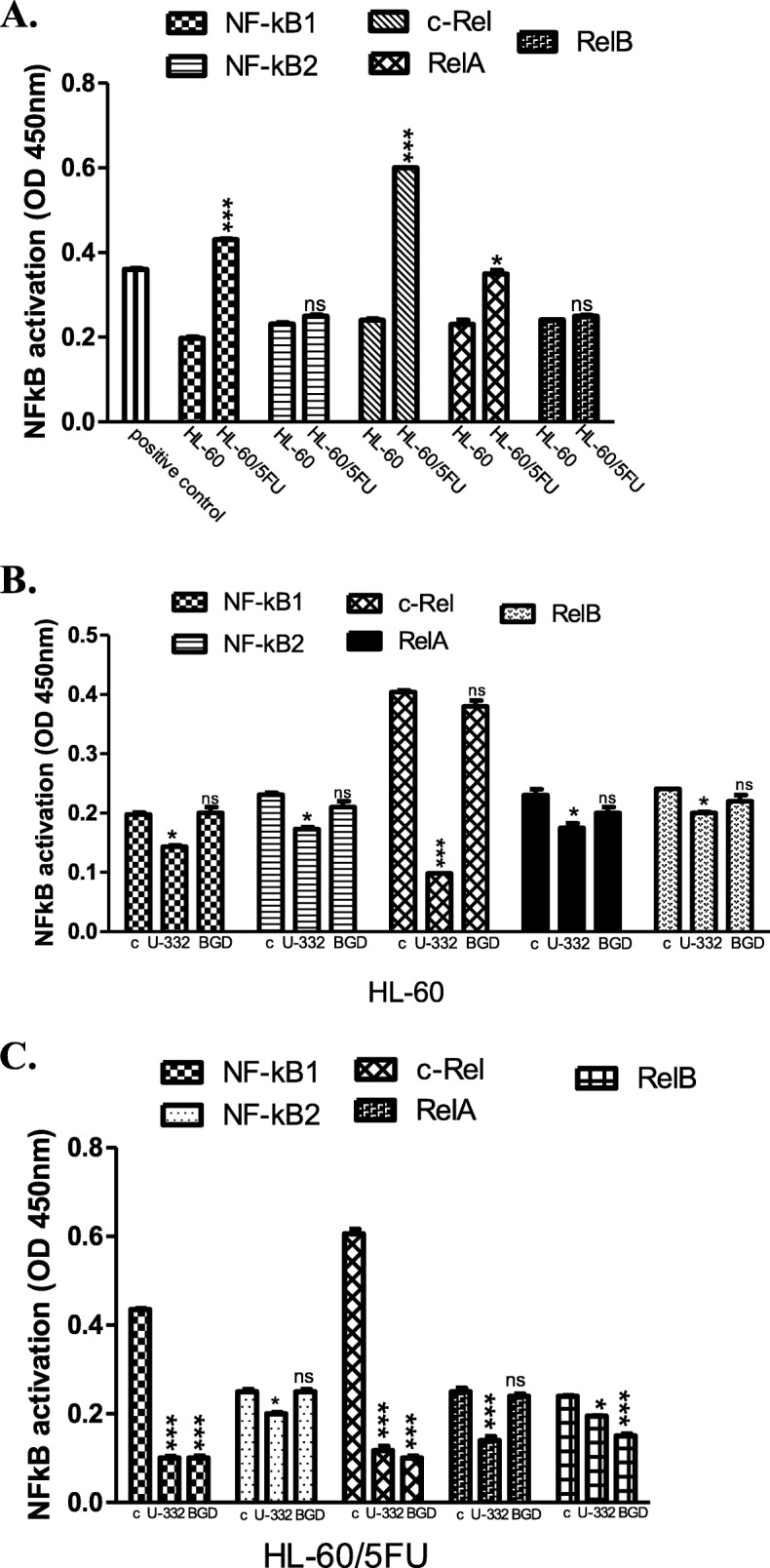
The activity of c-Rel, RelA, RelB, NF-κB1 (p100/p50), and NF-κB2 (p100/p52) in untreated HL-60 and HL-60/5FU (**a**); in HL-60 treated with U-332 or BGD (**b**); in HL-60/5FU treated with U-332 or BGD (**c**). Human ABC ELISA kits and extracts from cancer cells treated with U-332 or BGD (at IC_50_ concentration each) were used. As a positive control Raji nuclear extract was added. Data are expressed as mean ± SEM. Statistical significance was assessed using *test t* and one-way ANOVA and a *post-hoc* multiple comparison Student–Newman–Keuls test. **p < 0.01; ***p < 0.001 in comparison with control

Consistent with the enhanced gene expression, the activity of cRel, RelA and NF-κB1 subunits was significantly up-regulated in HL-60/5FU cells in comparison with the effect observed in HL-60 cells (Fig. 6a). Incubation of HL-60 cells with U-332 drastically reduced the activity of c-Rel, while BGD did not influence any of NF-κB subunits (Fig. 6b). In the resistant cell line, U-332 potently reduced activity of c-Rel, RelA and NF-κB1, while BGD exerted the strongest effect on c-Rel, RelB and NF-κB1 subunits (Fig. 6c).

## Discussion

Chemotherapeutic drugs are meant to kill disseminated cancer cells and prevent metastasis but many cancers develop resistance during treatment [36]. Activation of NF-κB or/and overexpression of ABC transmembrane proteins play a major role in the resistance of tumor cells to chemotherapy [2–4]. Many chemotherapeutic agents, including 5-FU, doxorubicin, etoposide or cisplatin have been reported to activate NF-κB and to up-regulate expression of ABC transporters [37–40]. In several cancer cell lines the inhibition of either NF-κB or ABC transporter activity increased intracellular accumulation of chemotherapeutic drugs [41–43]. For example imatinib, a known NF-κB inhibitor, reversed the acquired resistance to doxorubicin by down-regulating the level of ABCB1 through the inhibition of RelA (p65) activity [44]. Therefore, the combination of anticancer drugs with NF-κB and ABC transporter inhibitors can be considered an efficient approach to sensitize cancer cells to chemotherapy.

Various members of the NF-κB family are constitutively activated in many cancers via one of the two pathways: the canonical pathway involving RelA, NF-κB1 p50 and c-Rel and the non-canonical pathway engaging RelB and NF-κB2 p52. Generally, the canonical NF-κB pathway is known to mediate mostly inflammatory responses, while the non-canonical NF-κB and its components have been shown to have pro-tumorigenic effects in many cancer types. However, there is significant cross-regulation between the components of these pathways, emphasizing the importance of the NF-κB as a single, highly complex system with disease relevance in many types of cancer [45, 46].

In this report, we have shown that in HL-60/5FU resistant cells all 5 NF-κB subunit genes (*RelA, RelB, NF-kB1, NF-kB2* and *cRel*) were significantly up-regulated. By monitoring activation of both, the non-canonical and the canonical NF-κB pathway members we have demonstrated that in HL-60/5FU resistant cells U-332 was involved in the down-regulation of the canonical pathway (RelA, c-Rel and NF-κB1), while BGD inactivated some subunits of both pathways (c-Rel, RelB and NF-κB2). BGD is usually considered a universal inhibitor of both NF-κB pathways. Here, we have shown that in leukemic HL-60/5FU cells the levels of only three out of 5 NF-κB subunits were affected by BGD.

## Conclusions

The important finding of the research presented here and in our previous papers [31, 33] was the identification of the novel uracil analog as a potent inhibitor of NF-κB and ABCC1 transporter expression. The inactivation of NF-κB subunits was shown to correlate with the inhibition of ABC transporter activity which may lead to the increased accumulation of a drug in cancer cells.

## Data Availability

All data generated or analyzed during the present study are included in this published article.

## Abbreviations

5-FU: 5-Fluorouracil
ABC transporters: ATP-binding cassette transporters
ABCB1: P-glycoprotein
ABCC1: Multidrug resistance associate protein 1
ABCG2: Breast cancer resistance protein
AML: Acute myeloid leukemia
BGD: Bengamide
DMSO: Dimethyl sulfoxide
ECACC: European collection of cell cultures
FBS: Fetal bovine serum
HRP: Horseradish peroxidase
MDR: Multidrug resistance
NF-κB: Nuclear factor kappa-light-chain-enhancer of activated B cells
PCR: Polymerase chain reaction
TNFRs: Tumor necrosis factor receptors
